# Measurements of bone tunnel size in anterior cruciate ligament reconstruction: 2D versus 3D computed tomography model

**DOI:** 10.1186/s40634-014-0002-0

**Published:** 2014-06-26

**Authors:** Bernardo Crespo, Cathrine Aga, Katharine J Wilson, Shannon M Pomeroy, Robert F LaPrade, Lars Engebretsen, Coen A Wijdicks

**Affiliations:** Steadman Philippon Research Institute, 181 W. Meadow Dr. Suite 1000, Vail, 81657 CO USA; Department of Orthopaedic Surgery, Oslo University Hospital and Faculty of Medicine, University of Oslo, Oslo, Norway; The Steadman Clinic, Vail, CO USA; Oslo Sports Trauma Research Center, Oslo, Norway; Department of Biomedical Engineering, Steadman Philippon Research Institute, 181 W. Meadow Dr. Suite 1000, Vail, 81657 CO USA

**Keywords:** Anterior cruciate ligament, Tunnel enlargement, Revision ACL, 3D CT model

## Abstract

**Background:**

Revision anterior cruciate ligament (ACL) reconstruction requires a precise evaluation of previous tunnel locations and diameters. Enlargement of the tunnels, despite not usually affecting primary reconstruction outcomes, plays an important role in revision ACL management. Three dimensional (3D) computed tomography (CT) models are reported to be the most accurate method for identifying the tunnel position and possible conflicts with a revision tunnel placement. However, the ability of 3D CT to measure the tunnel size is still not proven. The goal of this study was to evaluate the ability of measuring the size of the bone tunnels in ACL reconstructed knees with 3D CT compared to the traditional two dimensional (2D) CT method.

**Methods:**

Twenty-four patients had CT scans performed immediately following ACL reconstruction surgery. Their femoral tunnels size were measured by a standard 2D CT measurement and then compared with three novel 3D CT measuring methods: the best transverse section method, the best fit cylinder method and the wall thickness method. The drill size used during surgery was used as a control measure for the tunnel width. Intra-class correlation coefficients were obtained.

**Results:**

The intra-class correlation coefficient and respective 95% confidence interval range (ICC [95%CI]) for the three methods compared with the drill sizes were 0.899 [0.811-0.947] for the best transverse section method, 0.745 [0.553-0.862] for the best fit cylinder method, −0.004 [−0.081 to −0.12] for the wall thickness method and 0.922 [0.713-0.97] for the 2D CT method. The mean differences compared to the drill size were 0.02 mm for the best fit transverse section method, 0.01 mm for the best fit cylinder diameter method, 3.34 mm for the wall thickness method and 0.29 mm for the 2D CT method. The intra-rater agreement (ICC [95%CI]) was excellent for the best transverse section method 0.999 [0.998-0.999] and the 2D CT method 0.969 [0.941-0.984].

**Conclusions:**

The 3D best transverse section method presented a high correlation to the drill sizes and high intra-rater agreement, and was the best method for ACL tunnel evaluation in a 3D CT based model.

## Background

Tunnel enlargement after an anterior cruciate ligament (ACL) reconstruction is a well-known phenomenon, noticed first in the early 1990’s following allograft reconstruction [1], but is also seen with different graft techniques and fixation methods [1–3]. Although no significant correlation between tunnel enlargement and clinical outcomes has currently been reported [4–9], tunnel widening may have serious implications for patients requiring ACL revision surgery. Revision ACL rates range from 10-25% [10], and a reliable assessment of the tunnel width and position is crucial to surgical planning [11].

Different methods for measuring tunnel width have been described in the literature using two dimensional (2D) radiography, computed tomography (CT) and magnetic resonance imaging (MRI) [12–16]. The 2D CT has been reported to be the best method for identifying the tunnels, especially immediately after surgery [12,17]. Other advantages of CT scans are that they eliminate the scaling issues present in plain radiographs and that they appear to be less affected by geometric factors that may influence the tunnel measurements due to knee positioning during image acquisition. Although providing the most accurate measurements, CT scans also have limitations. Interobserver and intraobserver reliability have been reported to be inconsistent [12,17].

Three dimensional (3D) computed tomography models have been developed to create an accurate 3D model of the bones using imaging software. These models have been largely used to investigate tunnel position following cruciate ligament reconstruction and reportedly have increased intraobserver reliability [18–20]. Additionally, 3D CT models were reported to be the most reliable imaging method of showing conflict between pre-existing and desired femoral tunnel locations prior to ACL revision surgery [21]. However, to the authors’ knowledge, 3D CT methods for evaluating tunnel width have not been studied to date.

The purpose of this study was to evaluate the accuracy of three novel methods for measuring tunnel width based on the 3D CT bone model of ACL reconstructed knees. The novel methods were compared to the current clinical method of measuring tunnel width using 2D CT images. We hypothesized that the novel CT bone model methods would be more accurate for measuring tunnel width than the current standard methods.

## Methods

### Sample selection

Prior to initiation, the study protocol was approved by the Institutional Review Board of the (Regional Committee for Medical Research Ethics) and a signed informed consent for release of scans was received from all participants. The patients’ selection criteria were: age between 18 and 40 years, 3 months of rehabilitation prior to the surgical procedure and a complete ACL tear verified by history, physical examination and confirmed by an arthroscopic procedure. Patients were excluded from the study if they presented with ACL revision reconstruction, ACL lesion of the contralateral knee, concomitant PCL, lateral or medial instability at the time of the surgery, established osteoarthritis with Kellgren-Lawrence classification grades 3 or 4 or hamstring grafts unable to have a minimum diameter of 5 mm for each bundle. The 24 patients enrolled into the study were randomized to single-bundle (SB) or double-bundle (DB) ACL reconstruction, with 12 patients in each group.

### Imaging protocol and 3D modeling

The CT scanner used was a Philips Brilliance 16-slice scanner, and the imaging specifications used for all patients included 1.5 mm slice thickness, 0.75 mm slice increment, 120 kV, 250 mAs, 500 mm field of view, 512 × 512 resolution. All patients had CT scans performed during the initial two days following surgery with their knee positioned in full extension. The high resolution images were evaluated by an independent investigator who was not involved in the surgical procedure or patient care.

**Figure 1:**
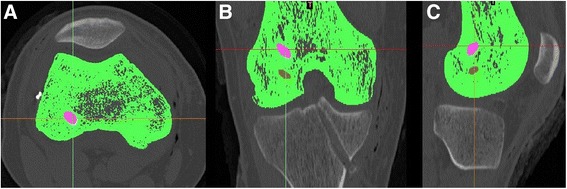
**Segmentation process of 2D CT images.** Segmentation process performed on the original 2D CT images viewed in axial **(A)**, coronal **(B)** and sagittal **(C)** CT images.

**Figure 2:**
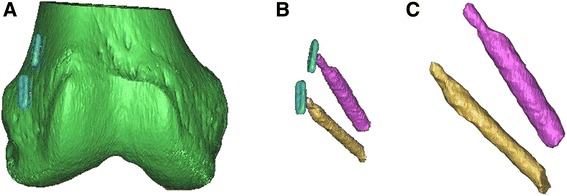
**3D model acquisition.** CT based 3D model showing all bone structures segmented **(A)**. Same image with the femur hidden **(B)**, and a closer view of the segmented femoral tunnels **(C)**.

The CT images were exported to an image analysis software (Mimics v1.6, Materialise, Leuven, Belgium) and a manual segmentation of the bone structures, bone tunnels and cortical buttons was performed. The segmentation process relies on using bone-soft tissue density variation on CT images, adjusting a density range to highlight bone anatomy on CT scan images. Manual revision of the CT images was performed to correct errors, and assure that the outline of the bone and tunnels were appropriately filled. This allowed for the creation of a patient-specific 3D bone model of the knee joint, with the tunnels appearing as empty spaces (Figures 1 and 2). This process has previously been validated, and has demonstrated high intraobserver and interobserver reliability and accuracy [22,23].

### Measurement methods

**Figure 3:**
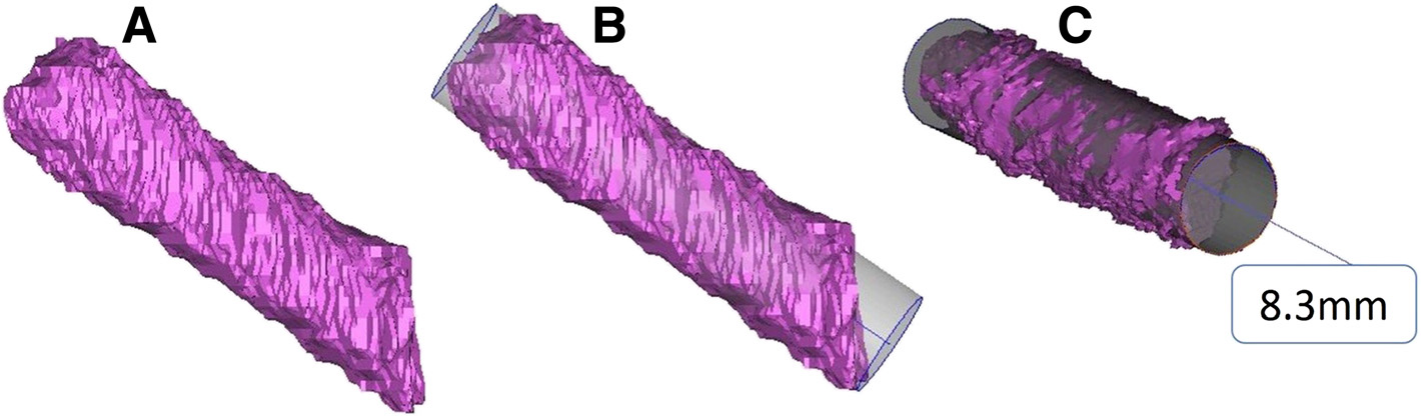
**Best fit cylinder method.** The original tunnel 3D model was exported to the 3D-matic® software **(A)**. An analytical cylinder was generated **(B)** and, a surface cylinder was created guided by the analytical cylinder. The cylinder diameter was measured **(C)**.

**Figure 4:**
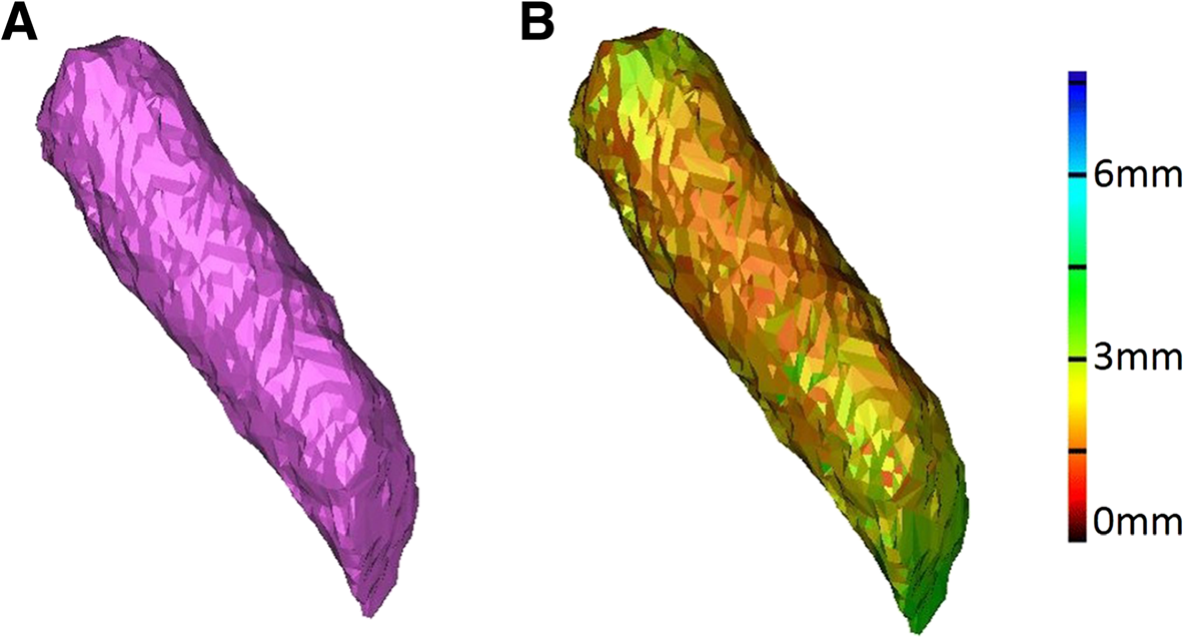
**Wall thickness method.** The original tunnel 3D model exported to the 3D-matic® software **(A)** and the Wall Thickness function was applied. A color scale showed the distance between the triangles and the opposite wall **(B)**.

**Figure 5:**
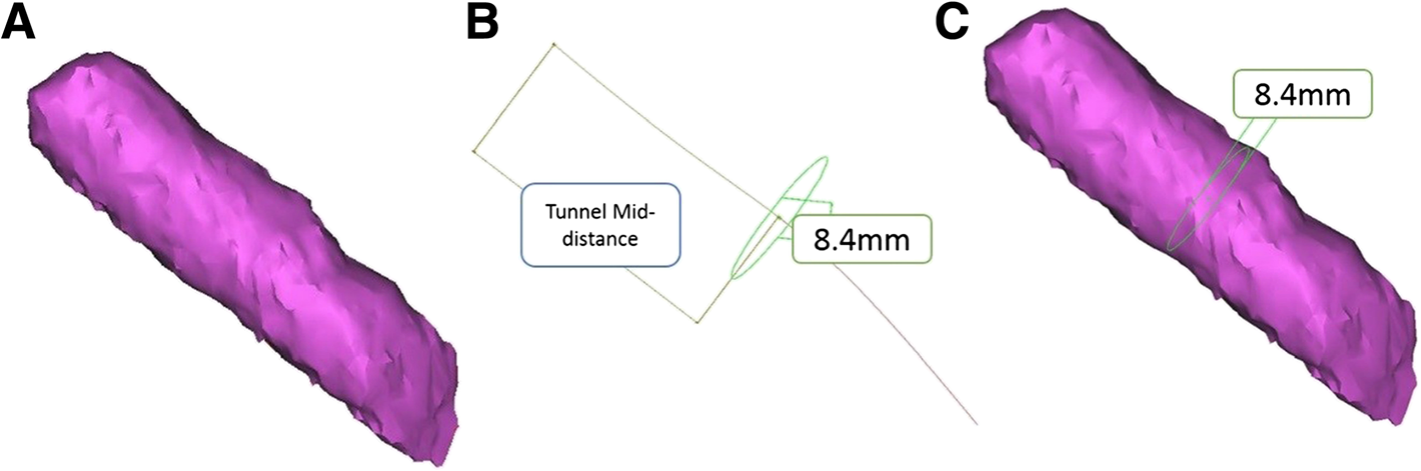
**Best transverse section method.** The segmented tunnel **(A)** was used as a guide for the automated centerline drawing made by the Mimics® software. Centerline half-way distances were measured and the best fit diameter on this points was evaluated **(B)**. Image of the tunnel model with the measurements on its surface **(C)**.

Using the 3D model of each patient’s knee, three different techniques for measuring tunnel width were evaluated: best fit cylinder, overall wall thickness and transverse section diameter. The best fit cylinder method consisted of creating an analytical best fit cylinder to the entire tunnel length (Figure 3). The wall thickness method consisted of an internal software function (Mimics, Materialise, Leuven, Belgium) that measured the perpendicular distance from each triangle that formed the 3D model to the opposite side of the model (Figure 4). The best fit transverse section method was performed by fitting a center axis to the entire tunnel length and then fitting a circle to the tunnel walls at its mid-length (Figure 5).

**Figure 6:**
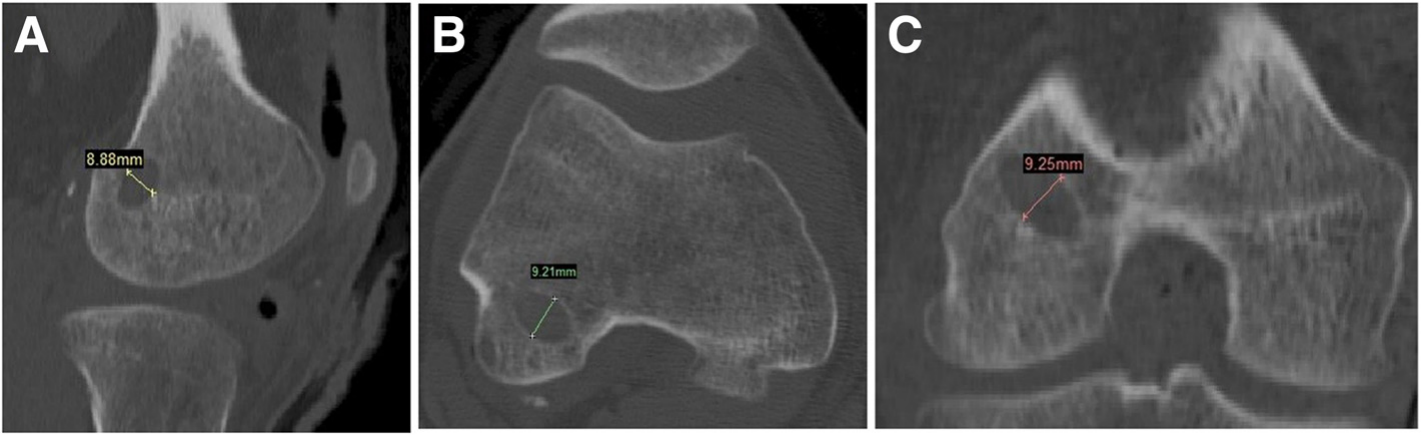
**2D CT method.** The measurements were made based on the original CT images, and the tunnels were assessed in all three CT planes: coronal **(A)**, sagittal **(B)**, and axial **(C)**.

The traditional method for measuring tunnel width in a 2D CT scan was also evaluated. The tunnel diameter was measured at the tunnel mid-point in all three image planes (axial, sagittal, and coronal) using a straight line drawing tool (Figure 6). The mean value of the measurements from the three image planes was used for comparison.

The 2D CT measurements and the best fit transverse section method measurements were repeated after one month to determine the intra-observer reliability of the method. The best fit cylinder and wall thickness methods were entirely automated by the software, having a complete agreement between the two consecutive measurements. An orthopedic surgeon (B.C.) trained by a musculoskeletal radiologist performed all measurements.

### Surgical technique

All procedures were performed by one surgeon (S.J.). The reconstructions relied on ACL anatomical landmarks, with the SB reconstruction targeting to have the tunnel in a central position of the ACL footprint, and the DB reconstruction aimed to place the tunnels in the center of each ACL bundle, as previously described by Muller et al. [24]. The drilling was performed through an accessory anteromedial portal with the knee in hyperflexion for both techniques, following a technique as described by Brown et al. [25]. The gracilis and semitendinosus tendons were harvested and doubled or tripled according to their size and length. The tunnel sizes were defined based on the graft diameters in a 0.5 mm increase graft ruler scale (Smith and Nephew, Andover, MA, USA), and the drill size was selected to match the graft diameter. A suspension device was used for femoral fixation (EndoButton - Smith and Nephew, Andover, MA, USA) and an interference screw (Biosure PK - Smith and Nephew, Andover, MA, USA), was used for tibial fixation.

### Statistical analysis

Because the tibia interference screw fixation method produced an intra-operative tunnel widening, and the tunnel size could be significantly different from the original drill size, the tibial tunnels were not used for comparison of methods. The measurements of the femoral ACL reconstruction tunnels were obtained by using the different techniques and were compared to the drill size used to prepare the respective tunnel. Mean (±SD) differences between measurement and drill size are reported. Intra- and inter-method agreement were assessed using the two-way random, single measures, absolute agreement form of the intra-class correlation coefficient (ICC). ICC values were classified as excellent (>0.75), fair to good (0.40 - 0.75) or poor (<0.40) [26–28]. All statistical calculations were performed using IBM SPSS Statistics, Version 20 (Armonk, NY, USA). Histograms of all measurements and differences from drill size were inspected and found to be reasonably normally distributed, prompting the use of parametric statistical tools.

## Results

### Tunnel size

**Figure 7:**
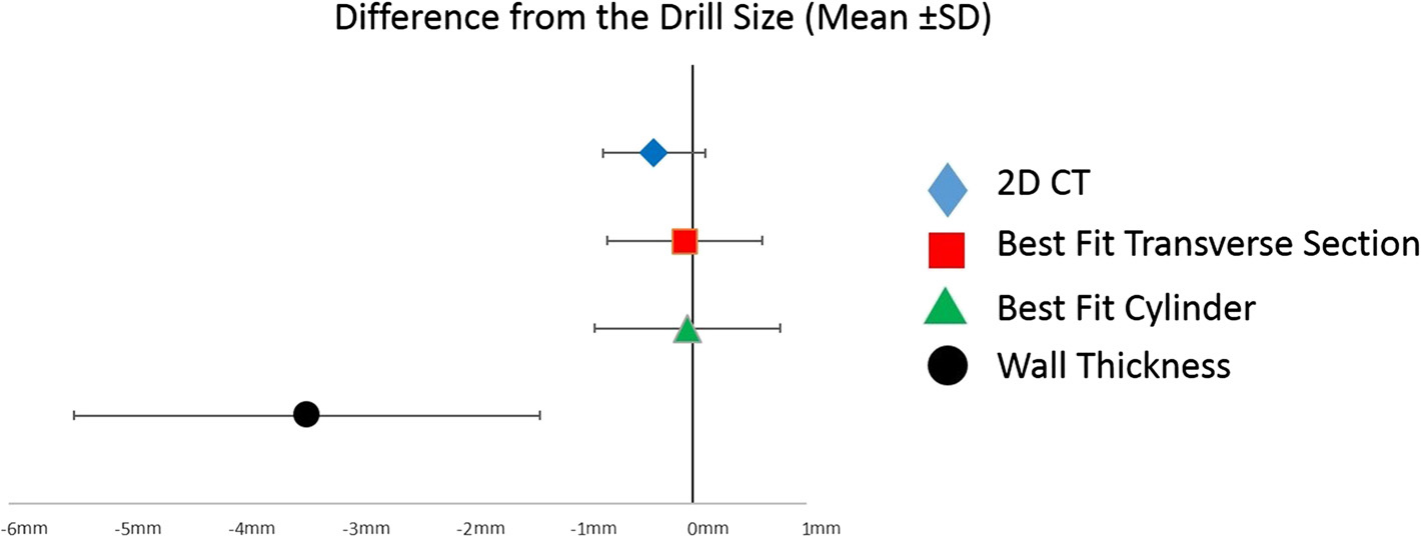
**Difference from Drill Size Mean.** Shows the difference between the average of the drill size and the mean of the measurements for each method with respective standard deviations.

The drill size range used for the ACL reconstructions in this study was from 5.0 to 9.0 mm and the difference between the average of the drill sizes and the measurements means and respective standard deviation were 0.29 ± 0.4 mm for the 2D CT method, 0.02 ± 0.6 mm for the best fit transverse section method, 0.01 ± 0.8 mm for the best fit cylinder diameter method and 3.34 ± 2.1 mm for the wall thickness method (Figure 7).

The intraclass correlation coefficient and respective 95% confidence interval range (ICC [95%CI]) comparing the measurements to the drill sizes was 0.922 [0.713-0.97] for the 2D CT method, 0.899 [0.811-0.947] for the best fit transverse section method, 0.745 [0.553-0.862] for the best fit cylinder method and −0.004 [−0.081-0.12] for the wall thickness (Table 1).

**Table 1:** **Methods agreement to the drill size**

**Absolute agreement with drill size**			
		**ICC** ^**α**^	**95% CI** ^**β**^
**2D CT**	**Mean**	0.922	[0.713 to 0.97]
	**Coronal**	0.876	[0.711 to 0.942]
	**Axial**	0.907	[0.548 to 0.968]
	**Sagittal**	0.876	[0.759 to 0.936]
**3D Model**	**Best fit transverse section**	0.899	[0.784 to 0.938]
	**Best fit cylinder**	0.745	[0.553 to 0.862]
	**Wall Thickness**	−0.004	[−0.081 to 0.12]

Shows the intraclass correlation coefficient (ICC) and respective confidence intervals range ([95%CI]) for the CT and 3D model first measurements compared to the drill sizes.

^α^ICC (intraclass correlation coefficient).

^β^95% CI (95% confidence intervals range).

Evaluating the measurements obtained on the original 2D CT scans, and comparing the measurements on each image plane (coronal, axial and sagittal) with the average of the drill sizes, there was an ICC [95%CI] of 0.922 [0.713-0.97] for the mean and 0.876 [0.711-0.942] for the measurements obtained on the coronal view, 0.907 [0.548-0.968] for the axial view and 0.876 [0.759-0.936] for the sagittal view (Table 2).

**Table 2:** **Intra-rater agreement on CT and 3D measurements**

**Intra-rater absolute agreement**			
		**ICC** ^**α**^	**95% CI** ^**β**^
**2D CT**	**Mean**	0.969	[0.941 to 0.984]
	**Coronal**	0.949	[0.903 to 0.974]
	**Axial**	0.947	[0.899 to 0.973]
	**Sagittal**	0.949	[0.903 to 0.974]
**3D Model**	**Best fit Transverse Section**	0.999	[0.998 to 0.999]

Shows the intraclass correlation coefficient (ICC) and respective confidence intervals range ([95%CI]) between the first and second measurements performed with the CT and 3D best fit circle methods.

^α^ICC (intraclass correlation coefficient).

^β^95% CI (95% confidence intervals range).

### Intra-rater ICCs

The intra-rater agreement (ICC [95%CI]) was excellent for the best-fit circle 0.999 [0.998-0.999] and the 2D CT method 0.969 [0.941-0.984]. The best fit cylinder and the wall thickness methods were totally automated, with total agreement (ICC of 1.00) between measurements.

## Discussion

The most important finding of our study was that the 3D best fit transverse section method presented excellent accuracy for measuring tunnel width and excellent intraobserver reliability. Of all the 3D model methods, the best fit cylinder method was the closest to the mean drill size. However, it had a higher standard deviation and lower accuracy, evaluated by the ICC, compared to the transverse section method. The wall thickness method produced significant smaller values than the mean drill size. Additionally, the 2D CT measurement method presented a high correlation to the drill sizes used to ream the tunnels and a high intraobserver reliability.

Several studies evaluated enlargement by comparing late post-operative CT images and the drill sizes used during the procedure. Comparing immediate postoperative images to the drill size diameters, we could access the accuracy of CT based methods. As expected, we obtained a high correlation between the drill diameter and the 2D CT measurements, validating the use of the drill size as the immediate postoperative diameter for the ACL femoral tunnels.

Although the 2D CT method for tunnel enlargement evaluation is commonly used, its limitations regarding alignment between CT plane cuts and limb/tunnel orientation have been previously described [17]. The ACL reconstruction technique has lately changed to a more anatomic graft placement with more oblique tunnels related to the femoral axis [29], and this technique change can theoretically increase the tunnel-CT axis orientation mismatch [14] attempted to minimize this factor by reorienting the images to align the tunnel axis, a step that has to be done for every tunnel and can be susceptible to error, especially in the presence of major enlargements, when the tunnel axis is less clear. In this study, the use of the average of all three 2D CT plane measurements improved the 2D method agreement when compared to the measurement in one single image plane (Table 2). However this strategy was time consuming compared to the 3D methods, once the 3D model is available, and hence not commonly used in clinical practice. Additionally, the tunnel enlargement does not seem to be an organized symmetric expansion of the tunnel, and Fink et al. [9] described that the tunnel enlargement differed according to the CT views used for the measurement, a 30.6% enlargement of the tibial tunnel, in the sagittal plane, against a 16.4% coronal plane enlargement was reported 2 years after surgery. All these issues can be potentially eliminated by the use of 3D model methods, because they portray the entire shape of the tunnel and thereby address the 3D geometry of the tunnel.

The best fit transverse section method presented an excellent agreement to the drill sizes (0.899 of ICC) and a mean difference of 0.07 mm to the drill sizes, comparable to 2D CT method with a 0.9222 ICC and 0.33 mm mean difference to the drill size. The intra-rater agreement was excellent (0.999 of ICC) because of the semi-automated nature of the measurement that only varied according to the position of the measurement. The ability of using the 3D image avoids the possible bias of the image cut selection and the tunnel-images mismatch present on 2D measurements. This method preserves the 2D CT method ability of evaluating the enlargement in different portions of the tunnel length. The best fit cylinder method presented a good agreement to the drill size (0.745 of ICC), and the closest mean difference to the drill size 0.04 mm. It has the advantage of evaluating the entire tunnel at once and was automated. The wall thickness method presented a poor agreement to the drill size and had a mean difference compared to the drill sizes of 3.38 mm due to variability in orientation of the triangles that composed the 3D model shape, downsizing the overall measurement.

A high intraobserver agreement value was obtained for the 2D CT method in our study. Previous papers reported much lower intraobserver agreement rates when using this method with ICC rates from 0.44 to 0.74 [5,17,30] This difference may be because our patients were evaluated immediately after surgery, when the tunnel remains cylindrical and has not enlarged. Measurement of an enlarged and misshaped tunnel on the 2D CT method would be more difficult, because it would greatly depend on how the enlargement was positioned within the CT field of view, if it was visible in the image planes and in which image the observer choose to perform the measurement. This could lead to a reduced reliability of the method.

3D CT scan is an important adjuvant in a clinical setting, and it has been validated to be the best method for tunnel placement evaluation and for planning a revision cruciate ligament surgery [18–20]. Many CT scanners currently have internal capacity of creating a 3D reconstruction of the bones and software to perform 3D measurements. Additionally this study demonstrated that with simple measurement tools an accurate measurement that addresses the 3D architecture of the tunnels was easily obtained.

This study has some limitations related to a small sample size, and the fact that one investigator performed all the measurements and an inter-observer evaluation was therefore not performed. The images were obtained in the immediate post operative and the effects of an asymmetrical enlargement in methods accuracy could not be evaluated. Additionally, the 2D CT measurements were collected in the original scan images, and the tunnels were not always well aligned to the images axis. However, this is the most usual measurement method on clinical practice, and no standardization for tunnel measurement is available in the literature.

## Conclusions

The 3D CT based best-fit transverse section measurement method demonstrated excellent accuracy and reliability for the ACL reconstruction tunnel measurement in immediate post-operative patients. This method was found to be the method of choice for tunnel measurement in 3D models. The best fit cylinder method also presented a high correlation to the drill size, and could be very helpful in revision ACL surgery planning by optimizing the new tunnel size to fit the previous tunnel. The wall thickness method presented poor results in evaluating the tunnels in the 3D model, and would not be recommended.

## Abbreviations

ACL: Anterior cruciate ligament
2D: Two dimensional
CT: Computed tomography
3D: Three dimensional
SB: Single-bundle
DB: Double-bundle
SD: Standard deviation
ICC: Intraclass correlation coefficient
CI: Confidence interval
